# Artificial intelligence in the tourism business: a systematic review

**DOI:** 10.3389/frai.2025.1599391

**Published:** 2025-07-25

**Authors:** Alexandra Lorena López-Naranjo, Mariana Isabel Puente-Riofrio, Verónica Adriana Carrasco-Salazar, Juan Diego Erazo-Rodríguez, Pamela Alexandra Buñay-Guisñan

**Affiliations:** ^1^Facultad de Ciencias Políticas y Administrativas, Universidad Nacional de Chimborazo, Riobamba, Ecuador; ^2^Facultad de Mecánica, Escuela Superior Politécnica de Chimborazo, Riobamba, Ecuador; ^3^Facultad de Ingeniería, Universidad Nacional de Chimborazo, Riobamba, Ecuador

**Keywords:** AI, tourism, optimization, technology, machine learning

## Abstract

In the tourism sector, AI has been gradually integrated to optimize operations, personalize customer experiences, and improve resource management, thereby transforming the way companies operate and connect with travelers. The aim of this re-search is to explore the application of AI in the tourism industry, identifying the main AI technologies used in the business, the specific areas or processes, their benefits, and challenges. For this purpose, a systematic literature review methodology was used, following PRISMA guidelines, from which 112 primary studies were obtained that contributed to answering the research questions. The main findings indicate that, in the tourism industry, the most commonly used AI technologies include Natural Language Processing (NLP) and deep learning with Neural Networks, with chatbots and models such as CNNs and LSTMs being particularly prominent. These technologies facilitate everything from the automation of interactions (such as bookings and customer service) to advanced data analysis for the personalization of services and strategic decisions, demonstrating their broad applicability and benefit in the sector. However, multiple challenges are also identified, ranging from high costs and advanced technological infrastructure to ethical and privacy concerns. Therefore, for proper implementation of AI in the tourism sector, it is crucial to carefully manage both the benefits and challenges to ensure its success.

## 1 Introduction

Since the beginning of the 21st century, the tourism industry has experienced rapid growth, becoming a central axis of economic development for many cities. These cities seek to enhance their overall economy through this activity (Wang, 2024a). This industry is highly interconnected and involves multiple sectors. Functionally, it is structured according to the theory of the five elements (eat, live, travel, shop, entertainment) and the four-point method, which includes tourism origin subsystems, destination, travel, and support. This interconnection promotes the development of sectors such as retail, food service, accommodation, and entertainment and even medicine, enhancing the tourist offering through the synergy between information, technology, capital, and labor, and forming an industrial conglomerate (Wei et al., 2021; Ashrafuzzaman et al., 2024).

Additionally, tourism is a dynamic and complex sector, influenced by changes in demand and tourist behavior, the emergence of new markets and players, and socioeconomic factors such as various crises. This makes decision-making, investment planning, and strategy creation in this field significant challenges (Savvopoulos et al., 2019).

Like all economic sectors, the tourism industry has undergone significant transformation with the integration of digital technologies. Among them, the Internet of Things, artificial intelligence, and data analytics have revolutionized both management and the experience of tourist destinations, driving the need for advanced technological tools (Srinivasan et al., 2025). Although the concept of smart tourism—which integrates functions such as data collection, resource management, product design, sales, tourist services, order management, and statistical analysis—has emerged, the actual implementation of these technologies is still limited, with few tourism enterprises fully adopting them (Gao et al., 2022).

Artificial intelligence (AI) technologies are transforming the tourism and hospitality industry, enabling machines to perform tasks previously done by humans, encompassing technologies such as machine learning and natural language processing, which facilitate travel planning and booking through recommendation systems based on the analysis of large volumes of data (El Hajal and Yeoman, 2024). These technologies help tailor services to the preferences and economic capabilities of travelers, overcoming informational and linguistic barriers (Pasichnyk and Artemenko, 2015).

In relation to previous research and the state of the art in the field, several fundamental studies have set significant guidelines. These works have explored various facets that relate artificial intelligence to the tourism sector. These studies provide a deeper understanding of the current technological capabilities and outline the path for future innovations in the field.

González-Mendes et al. (2024) conducted a bibliometric analysis to explore the state of the art of AI and its application in the hospitality and tourism industry from 1996 to January 2023, with the purpose of identifying research trends, opportunities, and challenges in this field. The main results indicate a growing adoption of AI technologies, which enhance operational efficiency, customer experience, and enable innovations in tourism management, especially in the context of the COVID-19 pandemic, which accelerated the implementation of technological solutions.

Puerta et al. (2024) conducted a study aimed at analyzing the impact of applied technologies in tourist risk management. They performed a literature review and bibliometric analysis of 128 documents indexed in scientific databases such as Scopus and Web of Science. The main findings reveal a consensus among authors on the relevance of technologies like Machine Learning, Artificial Intelligence, and Genetic Algorithms to enhance operational efficiency and competitiveness in the tourism sector. Additionally, the study highlights the low research output at the intersection of technology, risk management, and tourism, suggesting a need for more studies in this area to foster sustainable development of the sector.

On the other hand, the study by Mariani and Wirtz (2023) focuses on whether analytics have been defined precisely and consistently in hospitality management research and if cognitive analytics have been explicitly analyzed. The methodology employed consists of a systematic literature review (SLR) of articles published up to July 2022. The results reveal an exponential growth in the publication of articles on analytics in these fields, but they show that definitions of analytics are rarely specified and that cognitive analytics have not been properly labeled or analyzed in the current literature, suggesting a mismatch between data analysis practices and the terminology used by researchers.

Huang et al. (2021) investigate the integration of artificial intelligence (AI) applications in the hospitality and tourism industry, aiming to assess the adoption of 25 AI applications from the perspective of field experts. The main results indicate that AI applications offering personalized search and recommendations, as well as immersive experiences, are more likely to be adopted by consumers and workers. The study emphasizes the importance of considering attributes like relative advantage, complexity, and perceived risks in implementing these technologies in the tourism sector.

Although there is a growing number of studies addressing the application of artificial intelligence in the tourism sector, there are still significant questions that have not been fully explored. Research, such as that conducted by González-Mendes et al. (2024) and Puerta et al. (2024), has begun to unravel the impact and opportunities that artificial intelligence offers to enhance operational efficiency and customer experience in the hospitality and tourism industry. However, there are still less explored areas and open questions, such as what specific AI technologies are predominant in tourism, in which business areas they are most intensively used, and what are their specific benefits and challenges.

These gaps in the research suggest a need for deeper analysis that could include assessing how different AI technologies, such as machine learning, natural language processing, or recommendation systems, are applied in various aspects of tourism, from reservation management to the personalization of travel experiences. Additionally, it is crucial to understand the tangible benefits these technologies bring to the sector, as well as the potential challenges or barriers to their adoption. For this reason, the research through systematic literature review seeks to identify the following research questions:

- What are the artificial intelligence technologies present in the tourism industry?
- What business areas or processes does artificial intelligence optimize in the tourism industry?
- How does artificial intelligence optimize or benefit management in the tourism industry?
- What challenges do tourism companies face when implementing artificial intelligence technologies?

### 1.1 Theoretical framework

**Figure 1:**
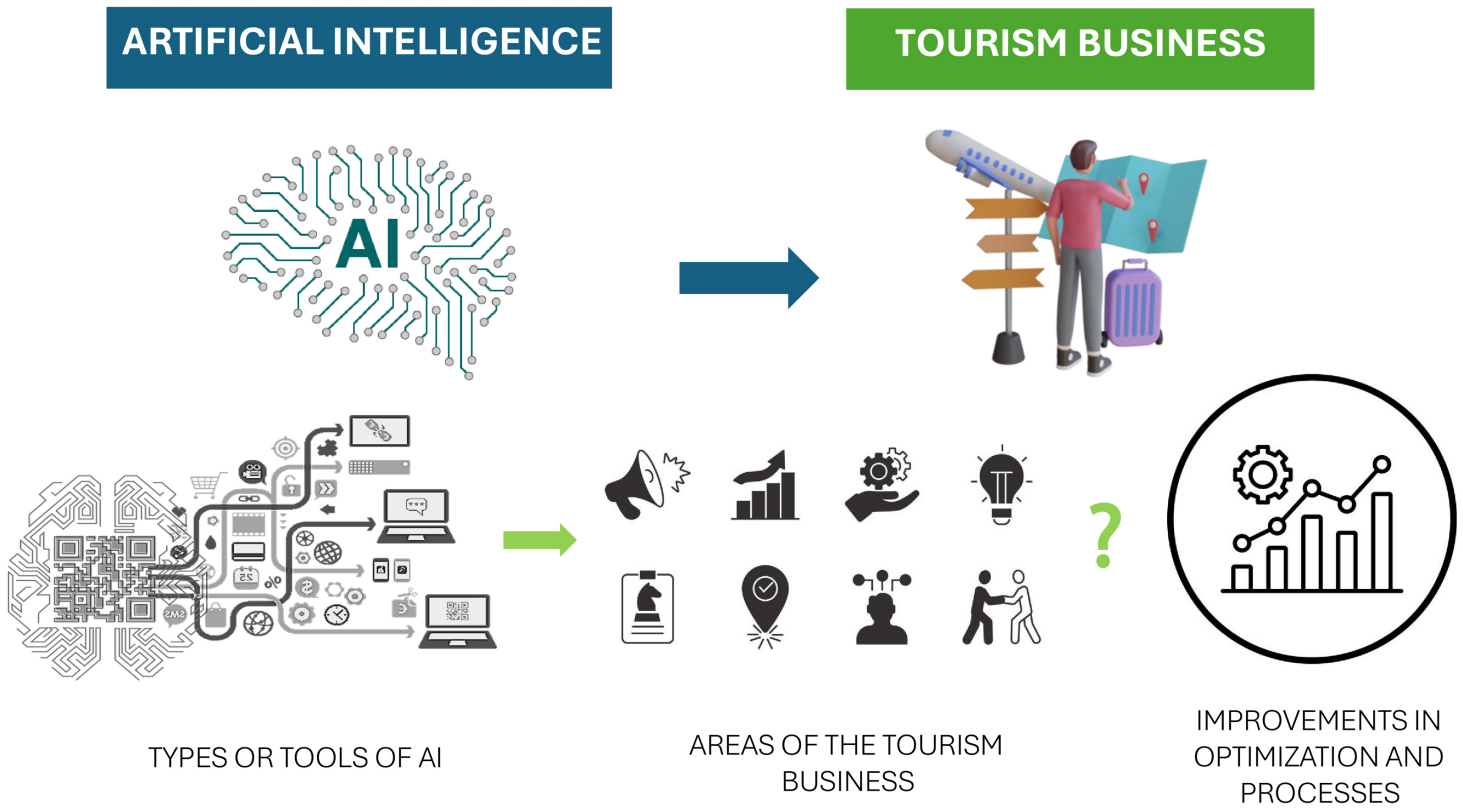
Map of the main variables of the study.

Figure 1 presents a diagram of the main variables of the study on the application of Artificial Intelligence (AI) in the tourism business, addressing three key aspects: first, the identification of AI, its types of technologies and tools; second, the application of these tools in various areas of the tourism business, such as marketing, customer management, and operational optimization; and third, the potential benefits and improvements in process optimization. This framework seeks to analyze how AI can transform the tourism industry by enhancing efficiency and strategic decision-making.

#### 1.1.1 Artificial intelligence

Artificial Intelligence (AI) aims to enable computers to perform tasks similar to human thinking, such as reasoning, which involves fundamental psychological processes like perception, association, prediction, planning, and motor control. Intelligence is not a singular concept but a complex set of interconnected abilities; similarly, AI uses various techniques to solve a wide range of tasks (Boden, 2017). According to Rouhiainen (2018), AI enables machines to use algorithms, learn from data, and apply that knowledge in decision-making, mimicking human abilities. Unlike humans, AI systems can process large volumes of information tirelessly and with fewer errors. Their learning and decision-making capabilities have grown exponentially.

Prominent applications of AI include robots and autonomous vehicles, facial recognition, natural language processing, and virtual agents, all of which enhance efficiency and user interaction across industries (Berente et al., 2021). Other key applications involve image recognition, business strategy optimization, predictive maintenance, object detection in autonomous vehicles, and cybersecurity. AI also supports business decision-making and automates tedious or hazardous tasks.

#### 1.1.2 Tourism business

The tourism business focuses on the creation and management of tourist destinations, which are specific places that offer unique experiences to visitors by combining resources, culture, infrastructure, and services (Alcocer, 2013). Fernandez (2018) states that tourism is a growing global phenomenon that generates divisions of opinion regarding its effects, being viewed by some as a driver of economic and social development, and by others as a source of exploitation and environmental degradation.

The management of tourism involves various organizational and strategic actions focused on achieving objectives through planning, organizing, leadership, and control. Additionally, the importance of implementing social and knowledge management is emphasized, which promote interaction between local actors and the transfer of experiences and resources, vital for the sustainable development of tourism activities in communities (Matos et al., 2022).

## 2 Materials and methods

The study is a systematic literature review that follows the PRISMA (Preferred Reporting Items for Systematic Reviews and Meta-Analyses) methodology, a structured approach that ensures transparency and rigor in the selection and analysis of scientific studies. PRISMA is based on four main phases: identification, screening, eligibility, and inclusion, which allows for the objective selection of the most relevant articles for the research topic (Page et al., 2021). This methodology contributes to the reproducibility and quality of the study, providing a comprehensive and well-founded overview of the impact of artificial intelligence on tourism.

### 2.1 Eligibility criteria and sources of information

The sources of information used in this research are SCOPUS and Web of Science, databases recognized for their wide interdisciplinary coverage and high scientific rigor, which ensures the quality and relevance of the selected studies. Regarding the inclusion criteria, an analysis period of the last 10 years was established, since artificial intelligence applied to tourism is a constantly evolving field, requiring the incorporation of updated literature. Publications in all languages were considered to provide a more global and comprehensive perspective. However, in the Web of Science database, emerging scientific journals were excluded, prioritizing those with greater impact and consolidation in the academic community. Additionally, errata and corrections were removed, ensuring the integrity and validity of the data analyzed in the systematic review.

### 2.2 Search strategy

**Figure 2:**
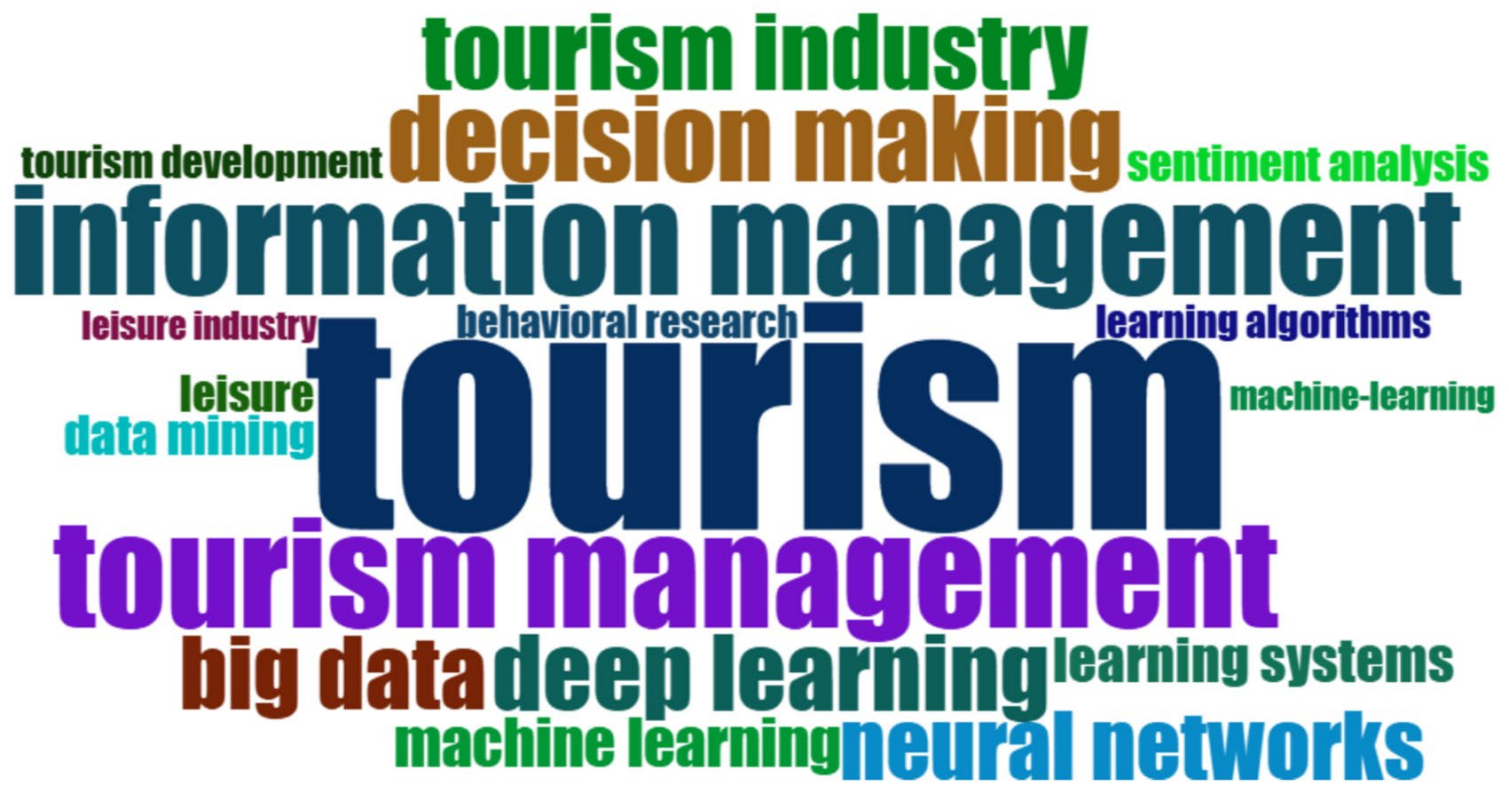
Keyword cloud.

The search strategy was carried out through an iterative and evidence-based process to ensure the inclusion of relevant studies. Initially, a preliminary search was conducted in the SCOPUS database, using a combination of key terms aligned with the research topic: TITLE (management) AND TITLE (tourism) AND TITLE-ABS-KEY (“artificial intelligence”). From the studies obtained in this initial search, additional terms were identified through keyword analysis with R Studio software, which allowed for detecting recurring concepts in the literature and optimizing the search strategy. This procedure was repeated twice, progressively refining the key terms to construct a robust final search string that ensured the collection of relevant and high-quality studies for the present systematic review. Figure 2 presents the word cloud with the main terms used in the search string.

In Table 1, the search strings used for each database are presented, along with the total number of studies. It is important to note that each database has different parameters and configurations for conducting searches, which implies the need to adapt the strategies to the specifics of each platform. These differences can include variations in the syntax of Boolean operators and options for filtering by thematic areas. Therefore, the construction of the search strings was carried out rigorously, ensuring methodological coherence and the acquisition of studies relevant for analysis.

**Table 1:** Search strings.

Database	String	Total
Scopus	(TITLE (management OR “decision making” OR “planning” OR “business” OR “administration”) AND TITLE (tourism) AND TITLE-ABS-KEY (“artificial intelligence” OR “AI” OR “neural networks” OR “deep learning” OR “machine learning”)) AND PUBYEAR > 2014 AND PUBYEAR < 2026 AND (EXCLUDE (DOCTYPE, “er”) OR EXCLUDE (DOCTYPE, “tb”) OR EXCLUDE (DOCTYPE, “bk”) OR EXCLUDE (DOCTYPE, “cr”))	150
Web of science	((TI = (management OR “decision making” OR “planning” OR “business” OR “administration”)) AND TI = (tourism)) AND ALL = (“artificial intelligence” OR “AI” OR “neural networks” OR “deep learning” OR “machine learning”)	69
		219

### 2.3 Study selection process and data extraction

**Figure 3:**
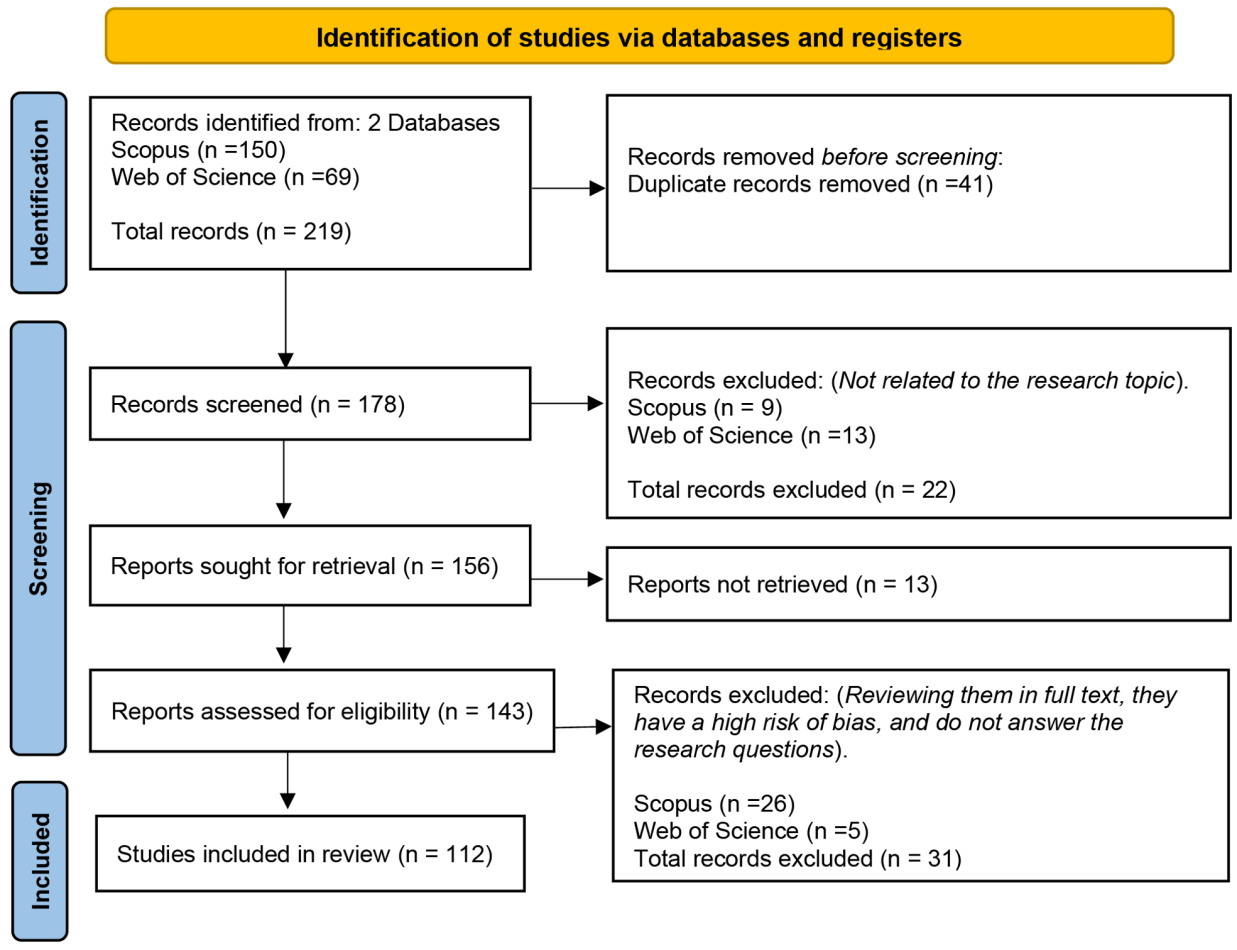
PRISMA flowchart.

For the selection of studies, a systematic process was carried out. Initially, the results obtained from the two databases used, SCOPUS and Web of Science, were grouped together, resulting in a total of 219 studies (see Figure 3). Subsequently, duplicates were removed (41 studies), reducing the number of documents to 178. Next, the authors conducted a detailed review of the title, abstract, and keywords of each study to discard those that deviated from the research topic, which led to the exclusion of 22 studies, leaving a total of 156. Of these, 13 studies could not be downloaded in full text, reducing the corpus to 143 studies. Finally, the selected documents were subjected to a bias risk assessment process, aiming to exclude those that presented a high risk of methodological bias. As a result, 112 primary studies were used for data extraction, from which the answers to the research questions posed in this study were obtained.

### 2.4 Bias risk assessment

A methodology adapted from the Cochrane strategy was used to assess the risk of bias in the studies, considering six fundamental criteria: random sequence generation, allocation concealment, blinding of participants and personnel, blinding of outcome assessment, completeness of outcome data, and selective reporting. This evaluation took into account the type of study, recognizing that not all criteria are relevant for each research. This process allowed for the identification of studies that presented an insignificant or minimal risk of bias, ensuring the reliability of the collected data.

**Figure 4:**
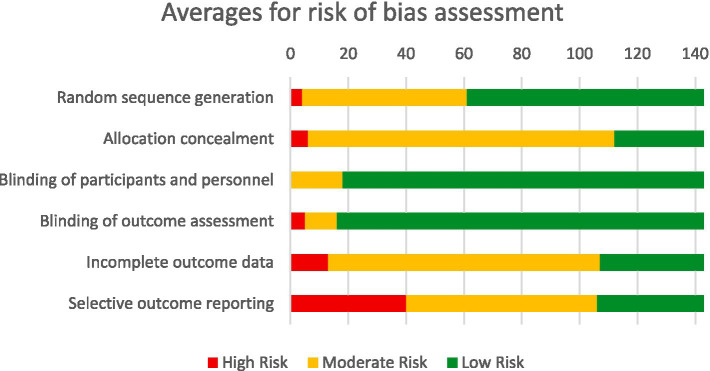
Bias risk assessment.

Figure 4 provides a graphical representation of the bias risk analysis, based on six specific criteria, applied to a total of 143 studies. This figure details the distribution of bias risk according to the evaluated criteria. Of these studies, 112 were classified as primary studies because they demonstrated a low risk of bias on average. For a more detailed understanding of the process and the results of the evaluation, Supplementary material can be consulted, where the full details of the analysis are provided.

### 2.5 Synthesis methods

For the processing of information, responses to the research questions from each analyzed study were extracted. It was observed that some responses were not explicitly mentioned, and in several cases, the studies presented similar information but expressed in different ways. To simplify and clarify the information for a more accessible presentation to the reader, the responses were categorized into different categories. This classification process and the methodology used for the synthesis of the information are described in the Supplementary material, allowing interested parties to thoroughly review the procedure and criteria used.

## 3 Results

Summary of the bibliometric data from the studies included in the systematic review, providing a statistical and descriptive basis about the consulted literature. Subsequently, the four research questions posed in the study are addressed in detail. The discussion begins with the artificial intelligence technologies used in the tourism sector, identifying specific areas of application within this sector. Next, the benefits that these technologies bring to tourism are explored, as well as the challenges they pose for their implementation and ongoing development.

### 3.1 Bibliometric data

**Figure 5:**
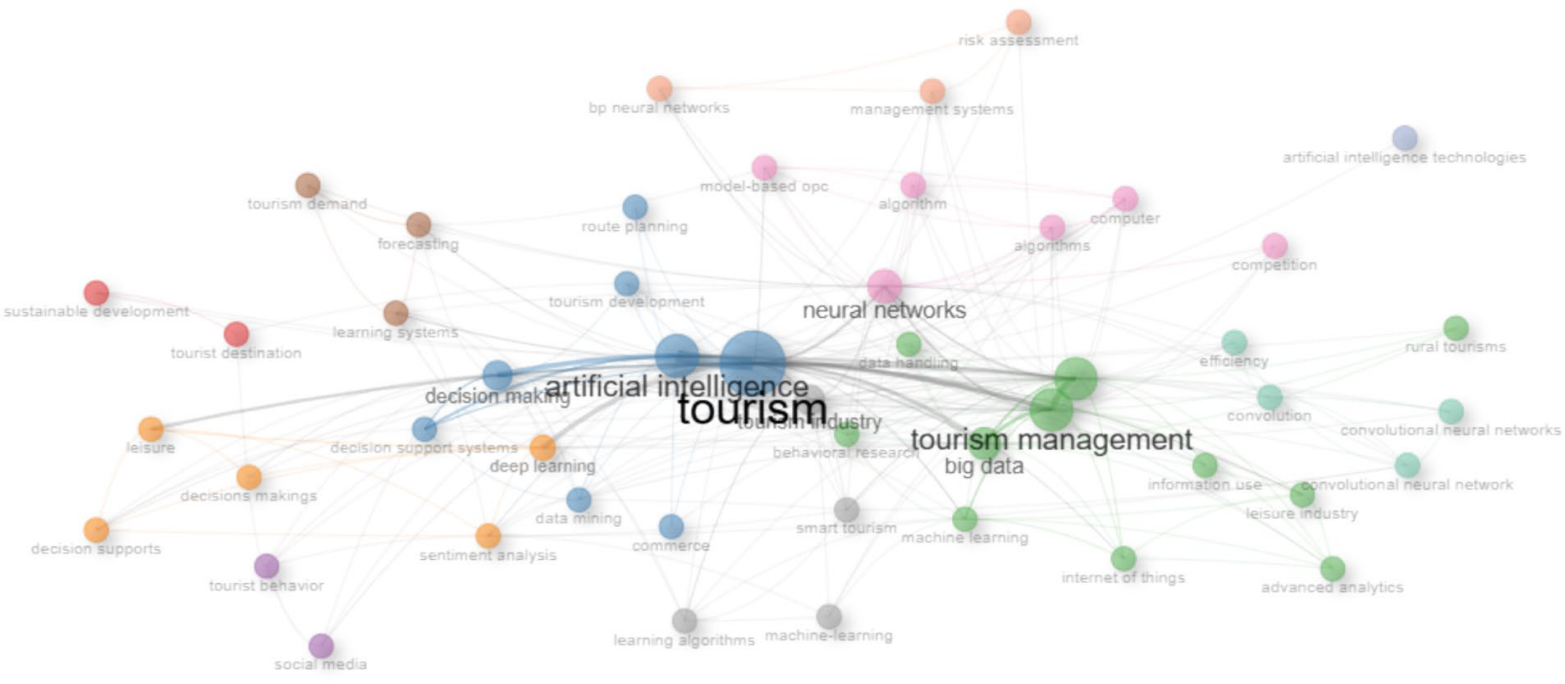
SLR keyword co-occurrence.

Figure 5 presents a keyword co-occurrence graph, in which the nodes and connections visualize the relationship between terms related to artificial intelligence and tourism. Different color groups are observed, indicating thematic clusters that group similar concepts. At the center, key terms such as “artificial intelligence,” “tourism,” and “management” are highlighted, which are larger, suggesting that they are the most recurrent and connected concepts within the field of study. The connections between the nodes indicate the frequency with which these terms appear together in scientific publications, reflecting the main research lines on the topic.

**Figure 6:**
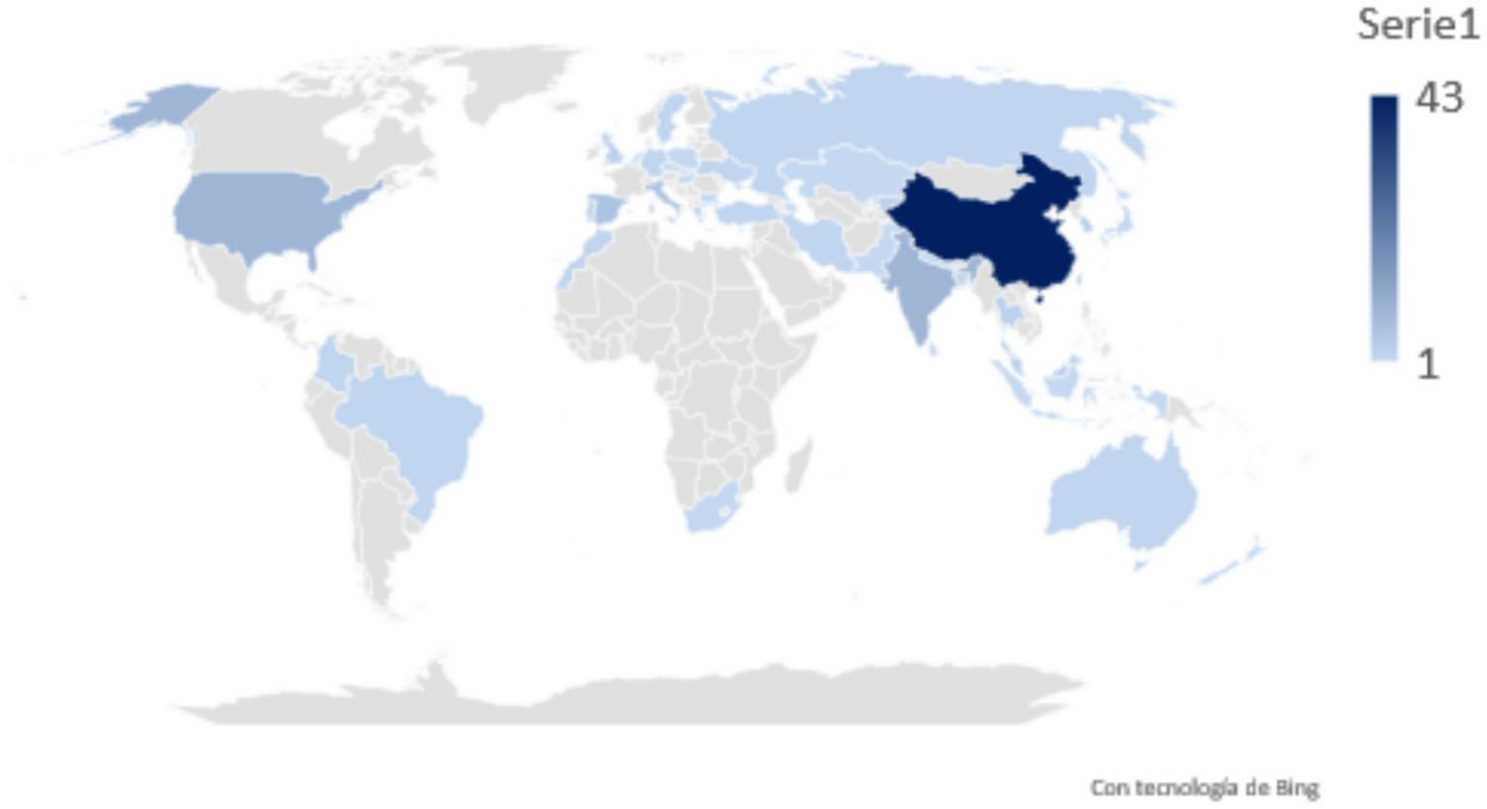
Geographic distribution of studies.

In the global landscape of research on technology and artificial intelligence, China stands out as a leader with a total of 43 studies, demonstrating its potential and strong interest in technological development (see Figure 6). This figure places China significantly ahead of other countries, with the United States and India each holding second place with 9 studies. They are followed by Spain and Italy with 6 studies each. Countries such as South Korea, Kyrgyzstan, Malaysia, Portugal, the United Kingdom, Thailand, and Taiwan have contributed with 3 studies each. Additionally, nations such as Germany, Australia, Bangladesh, Brazil, Colombia, Greece, Iran, Morocco, Russia, Sweden, Turkey, and Ukraine have contributed 2 studies each. Moreover, a diversity of countries including Azerbaijan, Belgium, Bulgaria, Cyprus, Slovakia, Hungary, Indonesia, Japan, Kazakhstan, Nepal, New Zealand, the Netherlands, Pakistan, Poland, the Czech Republic, Singapore, Sri Lanka, and South Africa have registered one study each, reflecting a broad geographical dispersion in interest and research in this field.

**Figure 7:**
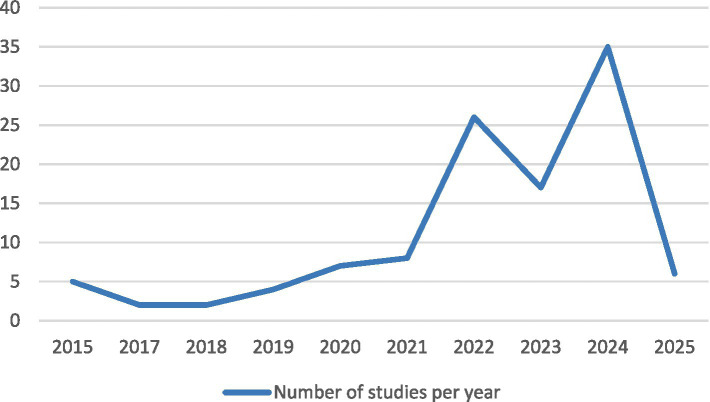
Yearly distribution of studies.

Figure 7 displays a graph representing the number of publications on artificial intelligence (AI) and tourism over the last 10 years. A progressive increase in the number of publications is observed, with a notable rise starting in 2021. It is evident that AI has experienced a significant surge in recent years, with 2022 and 2024 standing out as the years with the highest number of publications. The year 2025 shows a decline, which is due to the data only covering the month of January. However, the fact that there are already publications in such a short time suggests that 2025 will see an increase in research on AI and tourism, continuing the upward trend observed in recent years.

### 3.2 AI technologies in the tourism industry

Artificial intelligence (AI) technologies have the potential to profoundly transform the tourism industry, optimizing operations and enhancing customer experience while opening new business opportunities. AI enables advanced personalization of services, adapting to the specific needs and preferences of each user. In a global context increasingly focused on digitalization, understanding how AI integrates into tourism is important for industry professionals, urban planners, technology developers, and policymakers. Table 2 presents a summary of the technologies mentioned in the studies.

**Table 2:** AI technologies used in the tourism sector.

Type of AI technology	Algorithm/Method	Studies
Generative and natural language processing technologies	Chatbots	Dey and Shukla (2020), Tsang and Benoit (2020), Huang et al. (2021), Ansari et al. (2022), Chen and Xue (2022), Ivanov et al. (2022), Pradhan et al. (2022), Yuensuk et al. (2022), Abraham and Thazhathethil (2023), Kuo (2023), Çalışkan et al. (2023), Buitrago-Esquinas et al. (2024), Chen and Wei (2024), González-Mendes et al. (2024), Ku (2024), Ku and Chen (2024), and Nag and Mishra (2024b)
	Generative AI (ChatGPT)	Mariani and Wirtz (2023), Schuhbert et al. (2023), Buitrago-Esquinas et al. (2024), Christensen et al. (2024), Zhu et al. (2024), and Kim et al. (2025)
	Natural language processing (NLP)	Abeysinghe et al. (2018), Bustamante et al. (2020), Paolanti et al. (2021), Huseynova (2022), Ivanov et al. (2022), Yuensuk et al. (2022), Yu et al. (2022), Kuo (2023), Schuhbert et al. (2023), Zuo et al. (2023a), Ashrafuzzaman et al. (2024), Chen and Wei (2024), El Hajal and Yeoman (2024), Hu et al. (2024), Nag and Mishra (2024a, 2024b), Souha et al. (2024), Sánchez-Franco and Rey-Tienda (2024), and Wang (2024a)
	Attention-based models	Souha et al. (2024)
	Conditional random field (CRF)	Abeysinghe et al. (2018)
	Sentiment analysis and emotion detection	Abeysinghe et al. (2018), Yu et al. (2022), Ranga and Nagpal (2023), Schuhbert et al. (2023), Ashrafuzzaman et al. (2024), Nag and Mishra (2024b), Razali et al. (2024), Souha et al. (2024), and Rong et al. (2025)
	Labeled-LDA (Labeled Latent Dirichlet Allocation)	Yu et al. (2022)
Clustering	BIRCH for data clustering	Tzitziou et al. (2024)
	Fuzzy C-means clustering algorithm	Gao and Yan (2023)
	K-means algorithm	Dey and Shukla (2020)
Classification	Decision Trees	Silva et al. (2023) and Xu (2024)
	Support vector machines (SVM)	Bo and Shi-Ting (2015), Dey and Shukla (2020), Obogo and Adedoyin (2021), Wang (2022a), Silva et al. (2023), and Wu et al. (2023)
	Random forest	Chen et al. (2017), Bellodi et al. (2022), Mishra et al. (2024), Razali et al. (2024), and Xu (2024)
	Gradient boosting classifier (GBC)	Razali et al. (2024)
	Linear support vector machine (LSVM)	Razali et al. (2024)
	CART (classification and regression trees)	Wu et al. (2023)
	Naïve Bayes	Dey and Shukla (2020) and Yuensuk et al. (2022)
	Support vector regression (SVR)	Borrero et al. (2022)
	Fuzzy classification algorithms (not specified)	Wei (2022)
	Associative classification algorithms (not specified)	Pasichnyk and Artemenko (2015)
Association	Association rule algorithms (not specified)	Savvopoulos et al. (2019)
	Fuzzy association rule algorithms (not specified)	Wei (2022)
Neural networks and deep learning	Artificial neural networks (type not specified)	Abeysinghe et al. (2018), Ghaderi et al. (2018), Savvopoulos et al. (2019), Shi et al. (2019), Bustamante et al. (2020), Dey and Shukla (2020), Klimova et al. (2020), Tsang and Benoit (2020), Obogo and Adedoyin (2021), Yuensuk et al. (2022), Gao and Yan (2023), Hao and Zheng (2023), Khan et al. (2023), Schuhbert et al. (2023), González-Mendes et al. (2024), Ku and Chen (2024), Meng (2024), Nag and Mishra (2024a, 2024b), and Puerta et al. (2024)
	Back propagation neural networks (BPNN)	Bo and Shi-Ting (2015), Zhang et al. (2015), Li et al. (2019), Qian and Ge (2021), Gao et al. (2022), Wang and Wu (2022), Cai and Gunaban (2023), and Meng (2024)
	Convolutional neural networks (CNN)	Wei et al. (2021), Hou (2022), Li et al. (2022a), Lyu and Han (2022), Yang and Huang (2022), Yu et al. (2022), Zuo et al. (2023a), Meng (2024), Wang (2024b), and Srinivasan et al. (2025)
	Bidirectional long short-term memory networks (BiLSTM)	Meng (2024)
	Radial basis function networks (RBF network)	Hou and Wang (2024) and Zhang and Li (2025)
	Deep neural networks (DNN)	Kim et al. (2017), Shi et al. (2019), Dey and Shukla (2020), Paolanti et al. (2021), Li (2023), and Razali et al. (2024)
	Long short-term memory (LSTM)	Bellodi et al. (2022), Li (2023), Neshat et al. (2024), Qi and Han (2024), Razali et al. (2024), Souha et al. (2024), and Jung et al. (2025)
	Long-term recurrent neural networks (LSTM)	Li et al. (2022a), Meng (2024), and Srinivasan et al. (2025)
	Nonlinear autoregressive networks (NAR)	Borrero et al. (2022)
	Reinforcement learning algorithms (Deep Q-Learning)	Wang (2022b) and Rong et al. (2025)
	Multilayer perceptron (MLP)	Ghaderi et al. (2018), Siroosi et al. (2020), Li (2023), Razali et al. (2024), and Yang et al. (2024)
Optimization	Particle swarm optimization (PSO)	Zhang et al. (2015), Gao et al. (2022), Song and Xu (2023), and Meng (2024)
	Alternating direction method of multipliers (ADMM)	Yu and Wang (2022)
	Bee colony optimization algorithms	Gao and Yan (2023)
	Genetic algorithm	Pasichnyk and Artemenko (2015), Zhang et al. (2015), Gao and Yan (2023), and Puerta et al. (2024)
	Fastest virtual reality feature selection algorithm (FVR)	Zhang and Li (2025)
	Stochastic gradient descent (SGD)	Zhou et al. (2023), Razali et al. (2024)

Among the most mentioned technologies are those related to Natural Language Processing (NLP), Neural Networks, and Deep Learning, demonstrating their relevance in recent research. Within Generative and Natural Language Processing Technologies, Chatbots are the most supported technology in the literature. Among these, ChatGPT stands out as the most prominent generative artificial intelligence model. Developed by OpenAI, ChatGPT has gained wide recognition for its versatility in understanding and generating human-like text, supporting tasks such as content creation, language translation, virtual assistance, and strategic analysis. Its adaptability across various domains, including education, tourism, and customer service, underscores its role as a leading example of how generative AI can enhance decision-making, user engagement, and information accessibility in real time.

This demonstrates their growing application in automating human interactions through natural language processing and advanced conversational models. Natural Language Processing (NLP) also has a significant number of mentions, highlighting its fundamental role in language analysis and understanding in various contexts. In contrast, more specific approaches such as Attention-based models and the Conditional Random Field (CRF) algorithm have less presence in the literature.

In the field of Neural Networks and Deep Learning, there is a strong presence of references across various architectures. Artificial Neural Networks stand out, evidencing their versatility in multiple applications. Additionally, models of Convolutional Neural Networks (CNN) and LSTM (Long Short-Term Memory) networks also receive extensive support in the literature, confirming their importance in the analysis of sequential data and computer vision. Other architectures like Deep Neural Networks (DNN) and Multilayer Perceptron (MLP) have a considerable presence in the literature, reinforcing their application in complex problems.

In the area of Classification, the most referenced algorithms are Support Vector Machines (SVM) and Random Forest. This indicates their popularity in classification tasks compared to other approaches such as Decision Trees and Naïve Bayes, which have less support in the literature. Regarding Optimization, the Particle Swarm Optimization (PSO) Algorithm and Genetic Algorithm stand out with multiple references, suggesting their utility in optimization problems in artificial intelligence.

### 3.3 Areas or processes optimized by AI in the tourism industry

**Figure 8:**
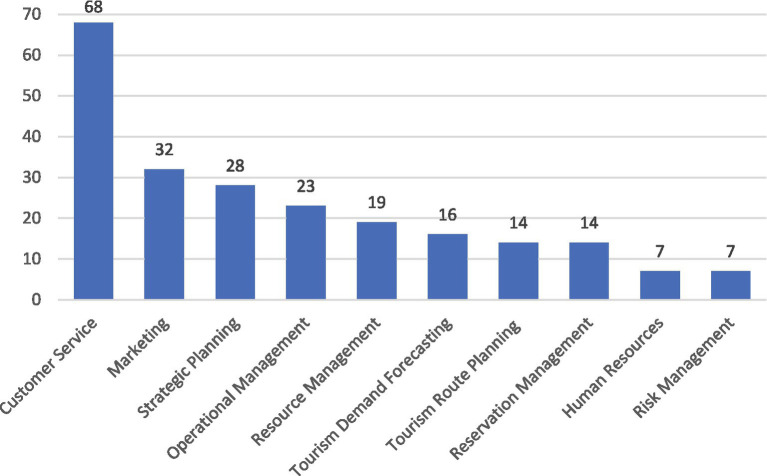
Main areas or processes utilizing AI in the tourism sector.

Artificial intelligence (AI) is revolutionizing the tourism industry by optimizing various processes and business areas. Figure 8 presents a classification of the areas or processes within the tourism sector where artificial intelligence (AI) is most utilized, ordered by the frequency with which the studies mention each area. This graph provides a clear view of the priorities and current trends in the application of AI in tourism, starting with the most frequently cited processes and descending towards those less mentioned.

One of the most frequently mentioned applications in the studies analyzed is customer service, where AI significantly enhances the tourist experience. This is achieved through chatbots that provide immediate responses to inquiries, facial recognition systems for faster and more efficient check-ins at hotels and airports, and virtual assistants that offer recommendations and personalized assistance during the trip.

The field of marketing is also frequently mentioned in the studies, where AI helps tourism businesses analyze large volumes of data to identify trends and consumer preferences, allowing for more precise segmentation and the development of personalized and effective marketing campaigns. Additionally, in strategic planning, AI contributes to resource optimization and decision-making improvement through predictive analysis and modeling of future scenarios, resulting in more efficient resource allocation and greater adaptability to market changes.

AI tools such as GPT and large language models (LLMs) can support the design and evaluation of tourism development plans by processing vast datasets, generating strategic insights, and facilitating scenario simulations. These technologies can be integrated with SWOT (Strengths, Weaknesses, Opportunities, and Threats) matrices to enrich strategic diagnosis and assist in identifying critical success factors. Moreover, when combined with methods such as the Analytic Hierarchy Process (AHP), AI can help build goal hierarchies, prioritize strategic objectives, and construct comprehensive decision trees that align with long-term development goals.

In operational and resource management, AI facilitates the automation of routine tasks and the optimization of resource allocation, such as staffing based on projected demand. Also in human resource management, AI can be used for staff selection, virtual reality-based training, and performance evaluation through algorithms that analyze employee effectiveness and efficiency.

AI also plays a crucial role in reservation management and predicting tourist demand, where predictive algorithms analyze booking patterns and market trends to adjust prices in real-time and maximize occupancy and revenue. Regarding the planning of tourist routes, trips, and activities, personalization is key; AI allows for the creation of customized itineraries that match the specific preferences and needs of each tourist, thus enhancing the overall travel experience.

Another highlighted process is also risk management, where AI helps identify and assess potential risks, from adverse weather conditions to socio-political changes in tourist destinations, allowing companies to take proactive measures to mitigate negative impacts on their operations and the safety of tourists.

Additionally, there are other processes mentioned to a lesser extent in the studies, such as environmental sustainability, where it helps monitor and manage the ecological impact of tourism activities. Financial management, where AI optimizes resource allocation and improves cash flow predictions, is essential for effective economic planning. It also intervenes in the evaluation and management of investments for tourist development, facilitating the identification of more profitable and sustainable investment opportunities. In terms of conservation, AI contributes to the planning and execution of strategies that balance tourist development with the conservation of natural and cultural resources. In energy management, it implements systems that reduce consumption and costs, optimizing energy use in tourism facilities. Moreover, AI is instrumental in the planning and management of events, from logistics to the personalization of experiences for participants. Lastly, in cybersecurity, AI strengthens defenses against digital attacks, protecting sensitive customer data and business operations in the tourism sector.

### 3.4 Benefits of AI in tourism industry management

Table 3 presents the main benefits of using artificial intelligence (AI) technologies, classified by areas and processes optimized in the tourism sector. This organization specifically details how various AI applications, such as reservation automation, natural language processing, facial recognition, among others, are implemented to improve operational efficiency, personalize the customer experience, make predictive decisions, and much more. Specific references are included that demonstrate the frequency and impact of each technology in the sector, providing a clear view of the current state and trends in the integration of AI in tourism.

**Table 3:** Benefits of AI in the tourism business.

Areas	Optimized processes	AI used
Reservation and operations management:	Automation of reservations and check-ins.	Natural Language Processing, facial recognition, neural network chatbots, virtual assistants with generative AI (Dey and Shukla, 2020; Tsang and Benoit, 2020; Huang et al., 2021; Zhang et al., 2021; Borrero et al., 2022; Abraham and Thazhathethil, 2023; Ku, 2024; Sánchez-Franco and Rey-Tienda, 2024; Xu, 2024).
	Optimization of tourist flow management.	Random Forest, LSTM, clustering techniques (Bellodi et al., 2022; Tzitziou et al., 2024).
	Efficient management of demand and resources.	Machine Learning, predictive analysis, Deep Learning, neural networks, natural language processing, optimization algorithms (Li et al., 2019; Huang et al., 2021; Wei et al., 2021; Bellodi et al., 2022; Wang, 2022a, 2024a; Li, 2023; Schuhbert et al., 2023; Thumrongvut et al., 2023; Wu et al., 2023; Nag and Mishra, 2024a, 2024b; Jung et al., 2025).
	Predictive maintenance of facilities.	Machine learning, deep learning, neural networks (Nag and Mishra, 2024b; Wang, 2024a).
Personalization and customer experience:	Personalized recommendations for itineraries and services.	Chatbots, generative AI, machine learning, natural language processing, deep learning, neural networks, classification and clustering algorithms (Huang et al., 2021; Gao et al., 2022; Kuo, 2023; Zuo et al., 2023a; Ashrafuzzaman et al., 2024; Chen and Wei, 2024; Christensen et al., 2024; Ku, 2024; Nag and Mishra, 2024a; Zhu et al., 2024; Kim et al., 2025; Rong et al., 2025).
	Customization of experiences based on analysis of consumer behavior and preferences.	Generative AI ChatGPT, natural language processing, deep neural networks, decision trees, random forest, Deep Learning (Lisi and Esposito, 2015; Paolanti et al., 2021; Borrero et al., 2022; Schuhbert et al., 2023; Wu et al., 2023; Zhou et al., 2023; Chen and Wei, 2024; Christensen et al., 2024; Mishra et al., 2024; Wang, 2024a; Zhu et al., 2024).
	Chatbots for real-time support and overcoming language barriers.	Generative AI ChatGPT, chatbots (Tsang and Benoit, 2020; Huang et al., 2021; Ansari et al., 2022; Chen and Xue, 2022; Ivanov et al., 2022; Pradhan et al., 2022; Yuensuk et al., 2022; Abraham and Thazhathethil, 2023; Kuo, 2023; Çalışkan et al., 2023; Buitrago-Esquinas et al., 2024; Chen and Wei, 2024; González-Mendes et al., 2024; Ku, 2024; Ku and Chen, 2024; Nag and Mishra, 2024b).
Predictive analysis and decision making:	Prediction of tourist demand to improve planning and operational efficiency.	Natural language processing, Deep Neural Networks (DNN), Long Short-Term Memory (LSTM), particle swarm optimization (PSO), deep learning, decision trees, random forest (Bo and Shi-Ting, 2015; Pasichnyk and Artemenko, 2015; Yamaka et al., 2015; Li et al., 2019; Tsang and Benoit, 2020; Wei et al., 2021; Bellodi et al., 2022; Borrero et al., 2022; Gao et al., 2022; Wang, 2022a; Lan, 2023; Li, 2023; Wu et al., 2023; Nag and Mishra, 2024b; Skare et al., 2025).
	Evaluation of historical data to adjust pricing and understand consumer behaviors.	LSTM (Long Short-Term Memory), recurrent neural network, Decision Trees, Random Forests (Tsang and Benoit, 2020; Huseynova, 2022; Christensen et al., 2024; Sánchez-Franco and Rey-Tienda, 2024; Xu, 2024; Jung et al., 2025).
	Informed decisions in the management of cultural heritage and adaptations to climate change.	Machine Learning (Li et al., 2022b; Xiao et al., 2024).
Marketing and customer relationship management (CRM):	Market segmentation and targeted marketing based on detailed data analysis.	Recurrent Neural Network (RNN), Convolutional Neural Network (CNN), deep learning, Natural Language Processing (Stylos and Zwiegelaar, 2019; Bustamante et al., 2020; Paolanti et al., 2021; Zhang et al., 2021; Lan, 2023; Schuhbert et al., 2023; Razali et al., 2024; Wang, 2024a; Jung et al., 2025).
	Management and analysis of customer opinions and sentiments.	Neural networks, Natural Language Processing, deep learning, sentiment analysis (Kim et al., 2017; Abeysinghe et al., 2018; Paolanti et al., 2021; Pradhan et al., 2022; Meng, 2024; Razali et al., 2024; Sánchez-Franco and Rey-Tienda, 2024; Srinivasan et al., 2025).
Sustainability and environmental responsibility:	Mitigation of negative environmental impacts in tourist areas.	Decision trees and Support Vector Machines (SVM), neural networks, genetic algorithms (Khan et al., 2023; Silva et al., 2023; Zuo et al., 2023b).
	More sustainable and resilient tourism management.	BP neural network, genetic algorithms, and particle swarm optimization (Zhang et al., 2015; Tzitziou et al., 2024).
Innovation and product development:	Development of new tourist products and services based on trends and predictive data.	Particle swarm optimization algorithm (PSO), Deep Learning, convolutional neural networks (Yamaka et al., 2015; Paolanti et al., 2021; Gao et al., 2022; Yang and Huang, 2022; Zuo et al., 2023a).
	Dynamic evaluation of tourist attractions and satisfaction levels.	Virtual agents and chatbots, convolutional neural networks, natural language processing, Deep Learning (Ghaderi et al., 2018; Stylos and Zwiegelaar, 2019; Huang et al., 2021; Wei et al., 2021; Ansari et al., 2022; Li et al., 2022a; Yang and Huang, 2022; Yuensuk et al., 2022; Ranga and Nagpal, 2023; Zuo et al., 2023a; Souha et al., 2024).
Security and risk management:	Optimization of security assessments and management of risks associated with tourism.	Back Propagation Neural Network (BP), Particle Swarm Optimization algorithm (PSO), Deep Learning (Qian and Ge, 2021; Abraham and Thazhathethil, 2023; Song and Xu, 2023, 2023; Zuo et al., 2023b; Neshat et al., 2024; Qi and Han, 2024).
	Use of biometric data to enhance security and travel experience.	FVR algorithm (Fastest Virtual Reality), RBF Network (Zhang and Li, 2025).
Operational efficiency and cost reduction:	Reduction of operational costs through process automation.	Deep Q-Learning, facial recognition systems, Natural Language Processing algorithms, Machine Learning, neural networks, generative AI (Chen and Xue, 2022; Ivanov et al., 2022; Suanpang et al., 2022; Yang and Huang, 2022; Dalkıran, 2023; Ashrafuzzaman et al., 2024; González-Mendes et al., 2024; Zhang, 2024; Zhu et al., 2024).
	Improvements in staff selection and reduction of repetitive tasks.	Natural language processing, Q-Learning, fuzzy association rule algorithms, fuzzy classification algorithms, chatbots (Wang, 2022b; Wei, 2022; El Hajal and Yeoman, 2024; Hu et al., 2024; Ku and Chen, 2024).

### 3.5 Challenges of using AI in the tourism industry

The implementation of artificial intelligence (AI) in the tourism industry presents significant challenges ranging from technical and operational issues to ethical and cultural concerns. Each of these challenge categories requires detailed consideration to ensure successful integration of AI in this sector:

#### 3.5.1 Costs and infrastructure

Adopting AI in tourism involves high implementation and maintenance costs (Yamaka et al., 2015; Siroosi et al., 2020; Razali et al., 2021; Wei et al., 2021; Ku, 2024; Puerta et al., 2024; Zhang, 2024), as the technology involved is complex and advanced. Companies must make substantial investments in appropriate technological infrastructure, which is not always possible due to the lack of existing infrastructure that can support new technologies (Klimova et al., 2020; Siroosi et al., 2020; Suanpang et al., 2022; Wang, 2022b; Lan, 2023; Thumrongvut et al., 2023; Çalışkan et al., 2023). This translates into the need to create or significantly enhance technological infrastructure, which can be prohibitive for many organizations, especially small and medium-sized enterprises.

#### 3.5.2 Ethical and privacy aspects

The implementation of AI in tourism also raises significant ethical concerns, particularly related to the privacy and security of the collected data (Bustamante et al., 2020; Dey and Shukla, 2020; Tsang and Benoit, 2020; Zuo et al., 2023a, 2023b; Ashrafuzzaman et al., 2024; Chen and Wei, 2024; Zhang, 2024; Nag and Mishra, 2024a, 2024b; Wang, 2024a; Zhang and Li, 2025). Ethical dilemmas arise from the use and implications of AI technologies, including concerns about unemployment due to automation and the impact on traditional (Song and Xu, 2023; Christensen et al., 2024; El Hajal and Yeoman, 2024; Ku, 2024; Nag and Mishra, 2024a; Puerta et al., 2024). Protecting personal data is critical for maintaining consumer trust.

#### 3.5.3 Integration and operability

Integrating AI with existing processes represents a significant challenge due to the complexity of the technologies and the variability in legacy systems (Dey and Shukla, 2020; Obogo and Adedoyin, 2021; Ansari et al., 2022; Borrero et al., 2022; Gao et al., 2022; Meng, 2024; Qi and Han, 2024; Zhang, 2024). Difficulties in data collection and standardization (Schuhbert et al., 2023) directly affect the AI’s ability to function efficiently, requiring continuous technological updates (Abraham and Thazhathethil, 2023) and adaptations in the daily operations of tourism companies to keep up with technological advances.

#### 3.5.4 Data and analysis

The quality and accuracy of data are fundamental for the effective operation of machine learning models (Abeysinghe et al., 2018; Savvopoulos et al., 2019; Stylos and Zwiegelaar, 2019; Paolanti et al., 2021; Hu, 2022; Zyma et al., 2022; Silva et al., 2023; Wang, 2024a; Xu, 2024; Skare et al., 2025). Issues related to incomplete, inaccurate, or biased data can lead to erroneous conclusions or ineffective predictions. Moreover, AI depends on large volumes of data for its learning and optimization, which poses challenges in managing and analyzing these massive data sets (Razali et al., 2021; Bellodi et al., 2022; Yu, 2022; Song and Xu, 2023).

#### 3.5.5 Training and resistance to change

There is a natural resistance to change within organizations that can hinder the adoption of new technologies like AI (Stylos and Zwiegelaar, 2019; Gao et al., 2022; Meng, 2024). This resistance often stems from a lack of understanding of the technology and fear of the unknown. Intensive training is crucial to ensure that staff understand and embrace new technologies, but finding and developing the technical skills necessary to manage these advanced technologies is another significant challenge (Razali et al., 2021; Sifolo and Henama, 2021; Wei et al., 2021; Ansari et al., 2022; Wang, 2022a, 2022b; Meng, 2024; Puerta et al., 2024; Qi and Han, 2024).

#### 3.5.6 Reliability and accuracy of the technology

Limitations of AI in terms of contextual accuracy and depth of analysis are concerning (Kim et al., 2025). Variability and noise in data can affect the accuracy of predictions (Li, 2023), which in turn can lead to the generation of incorrect information, damaging consumer trust and the company’s reputation (Christensen et al., 2024; Zhu et al., 2024).

#### 3.5.7 Cultural and social

It is crucial to ensure that the implementation of AI does not alter the cultural authenticity of tourist destinations and the real interaction between humans (Boukherouk et al., 2020, 2020; Schuhbert et al., 2023; Nag and Mishra, 2024b). Additionally, the cultural and organizational adaptation to new technologies is a process that requires time and effort, and the acceptance of these advanced technologies by customers and employees is essential for their success.

Each of these challenges requires a well-thought-out and executed strategy to overcome them, ensuring that the technology is integrated in a way that benefits both businesses and consumers in the tourism sector.

## 4 Discussion

The integration of emerging technologies, particularly artificial intelligence (AI) applications, has been gaining traction in the tourism sector, demonstrating significant transformative potential. According to Ivanov et al. (2022), technologies such as automated check-ins at hotels and airports, as well as customer-centric solutions like chatbots and voice-activated systems, have been implemented to enhance both operational efficiency and customer satisfaction. These technologies enable smoother management and more personalized service, critical factors in the digitalization era. This information aligns with the technologies identified in the literature review.

In the realm of artificial intelligence, ChatGPT and other generative intelligence applications stand out as a relevant tool for the tourism industry, specifically in customizing customer service and enhancing user experience. According to Kim et al. (2025), tools like ChatGPT utilize natural language processing (NLP) to interact more fluidly and effectively with users (Kuo, 2023). ChatGPT has the potential to revolutionize the service model in the hospitality and tourism industry (Tuo et al., 2025). It enables faster and more autonomous travel decision-making for tourists, while enhancing service quality through user feedback mechanisms and emotional intelligence programming.

One of the most notable applications of neural networks is in predicting tourist demand. As highlighted by Tsang and Benoit (2020) and Hou and Wang (2024), these networks are capable of analyzing historical data and behavioral patterns to forecast tourist flows, enabling companies to adjust their operational and pricing strategies accordingly. Furthermore, neural networks also play a crucial role in customizing the customer experience. Through classification and clustering algorithms, these networks can analyze travelers’ preferences and offer personalized recommendations for itineraries, accommodations, and activities (Bustamante et al., 2020). This adaptability results in an experience more aligned with consumer desires, which in turn can increase customer satisfaction and loyalty.

Regarding benefits, one of the most notable contributions of AI is process optimization. For example, automating reservations and using customer relationship management (CRM) systems allows businesses to handle large volumes of transactions more efficiently (Stylos and Zwiegelaar, 2019). This not only saves time and resources but also reduces the likelihood of human errors in managing bookings and orders.

AI can also be integrated into strategic business planning and operations by enabling advanced forecasting models for customer demand and more effective revenue management strategies (Gursoy and Cai, 2025). Additionally, it can automate repetitive administrative tasks, improving operational efficiency (Wang et al., 2025). However, despite growing interest in AI applications, few studies have explored its potential to drive business development or streamline organizational management.

The customization of the customer experience is a major contribution of AI technologies, enabling businesses to analyze consumer behavior and offer personalized services, such as tailored itineraries and travel recommendations, which enhance satisfaction and brand loyalty. However, as noted by Christensen et al. (2024), yet, this innovation comes with practical challenges, including the risk of “AI hallucinations,” or the generation of inaccurate or misleading information. Such misinformation can undermine trust and pose significant risks to the tourism industry.

AI also enables the analysis of large data sets that help predict market trends and behaviors. Analytical models like deep neural networks can forecast tourist demand, helping businesses adjust their prices and offers based on more accurate predictions (Xu, 2024). This anticipatory capacity is crucial for maximizing profitability and operational efficiency.

In most studies analyzed, the incorporation of AI in the tourism industry brings various operational or financial benefits and also enhances the customer experience by offering more efficient personalization and care. However, to maximize these benefits, companies must address challenges related to implementation, such as data management and consumer acceptance.

### 4.1 Limitations and future work

While artificial intelligence (AI) and emerging technologies have the potential to revolutionize the way tourism operations are managed and services are delivered, their effective adoption depends on a deep understanding of the dynamics of the sector, consumer expectations, and the ethical context in which these technologies are implemented. An integrative approach that addresses these aspects will be crucial to maximize the effectiveness of the proposed solutions and ensure alignment with market demands.

The adoption of such technologies also raises concerns about job loss and the dehumanization of service, requiring companies to perform a detailed cost–benefit analysis before implementation (Ivanov et al., 2022). An important aspect that companies must consider is the ethical and social implications of automation in tourism, offering technological solutions that not only focus on efficiency but also align with customer expectations, needs, and trust.

It is also to acknowledge that most current AI models rely heavily on statistical patterns derived from large datasets, which, while effective for identifying trends and optimizing existing processes, may inadvertently overlook original or context-specific solutions. This limitation is particularly relevant in the field of tourism development, where innovative strategies such as collective strategic planning and the construction of multi-level objective trees play a crucial role. These approaches emphasize participatory decision-making and nuanced goal-setting that adapt to the unique social, cultural, and environmental contexts of cities, megacities, and countries. Therefore, while AI can enhance analytical capacity, its integration with human-centered and collaborative planning methodologies remains essential for generating holistic and sustainable development strategies.

Despite its benefits, the use of AI also presents environmental challenges, particularly due to the high energy consumption involved in training large language models. This process contributes to a significant technological carbon footprint, raising concerns about sustainability. As AI becomes more integrated into tourism planning, it is essential to balance innovation with environmental responsibility by promoting energy-efficient practices and aligning digital tools with sustainable development goals.

Therefore, for AI to be effectively implemented in the tourism sector, an approach that considers not just technological capabilities but also user experiences and concerns is essential. For the future, it is suggested that studies delve deeper into evaluating the effectiveness of artificial intelligence technologies in different segments of the tourism sector, such as hospitality, travel planning, and destination management. It is crucial to explore how AI integration can enhance sustainability in tourism, especially in a context of growing concern over environmental impact. Additionally, future research should compare the performance and applicability of traditional machine learning approaches versus large-scale models like LLMs, in order to better understand their respective advantages, limitations, and the contexts in which each offers the greatest value.

## 5 Conclusion

The study addresses the growing integration of artificial intelligence (AI) in the tourism industry and how this phenomenon has transformed both management and the customer experience. The results indicate an increasing adoption of AI technologies, which are enhancing operational efficiency and the customer experience, especially from the context of the COVID-19 pandemic, which accelerated the implementation of technological solutions.

Regarding AI technologies, it was identified that it encompasses a wide range of algorithms and specific applications according to the studies reviewed. In the realm of generative technologies and language processing, chatbots and generative models like ChatGPT stand out, which use natural language processing (NLP) and attention-based models to analyze sentiments and detect emotions, as well as for more specialized tasks such as clustering and classification. These studies employ everything from traditional algorithms like decision trees and support vector machines to more advanced techniques like random forest and gradient boosting classifier. On the other hand, neural networks and deep learning remain a crucial pillar in AI development. These technologies are applied in varied contexts that include data analysis, pattern recognition, and optimization, using methods like the particle swarm algorithm and genetic algorithms, demonstrating their versatility and capacity to adapt to different problems and sectors.

The implementation of artificial intelligence (AI) technologies has provided several benefits in the tourism sector, such as the management of reservations and operations which has significantly improved processes through the automation of bookings and check-ins using natural language processing, facial recognition, and virtual assistants. In terms of personalization and customer experience, personalized recommendations through chatbots and classification algorithms stand out, enhancing customization based on the analysis of consumer behaviors and preferences.

However, despite its potential to foster innovation and improve operational efficiency, the sector faces significant challenges, including resistance to adopting new technologies and an infrastructure often insufficient to support such changes. It was also evident that applications seeking to automate operations face greater resistance, highlighting the importance of considering factors such as relative advantage and perceived risks in the development and implementation of these technologies. These issues underline the need for an integrative approach to understanding and promoting the effective application of AI in this field.

### 5.1 Study limitations

Some inherent limitations of the study, which must be considered, include the dependence on bibliometric analysis which, while providing an overview of the evolution of research in artificial intelligence in the tourism sector, may not fully capture the diversity of approaches and specific contexts addressed in individual studies. Another limitation refers to the risk of methodological bias in the reviewed studies. These considerations should be taken into account for future complementary studies that include a wider variety of sources and approaches to obtain a more holistic understanding of the phenomenon in question.

## Data Availability

The original contributions presented in the study are included in the article/Supplementary material, further inquiries can be directed to the corresponding author.
